# Person‐Centered Care Planning: Palliative Symptom Management in Multiple Chronic Conditions

**DOI:** 10.1002/lrh2.70105

**Published:** 2026-07-26

**Authors:** Kim Kuebler, Julian Gallegos, Eduardo Bruera, Matthew Sorenson

**Affiliations:** ^1^ Multiple Chronic Conditions Resource Center Brunswick Georgia USA; ^2^ College of Health and Human Sciences, School of Nursing Purdue University West Lafayette Indiana USA; ^3^ Department of Palliative, Rehabilitation, and Integrative Medicine, Unit 1414 UT MD Anderson Cancer Center Texas USA; ^4^ Graduate Nursing Education Texas A&M University College of Nursing Bryan Texas USA

**Keywords:** multiple chronic conditions, palliative symptom management, person‐centered care

## Abstract

**Background:**

Multiple chronic conditions (MCC) drive high symptom burden and utilization of healthcare resources. Person‐centered palliative symptom management approaches are underused in primary care.

**Approach:**

Narrative synthesis of major policy recommendations and practice literature to propose a primary‐care‐implementable framework (screening, triage, and management using Edmonton Symptom Assessment System).

**Key Points:**

Early, protocolized symptom screening and management in MCC is associated with fewer exacerbations, hospitalizations, improved physical functioning, and quality of life; feasible to deliver by primary care teams with targeted training.

**Implications:**

Shift palliative skills upstream in primary care; adopt standardized tools; and measure quality outcomes.

*What has surprised me is how little palliative care has to do with death. The death part is almost irrelevant. Our focus isn't on dying. Our focus is on quality of life*. Balfour Mount MD, McGill University

## 1 Introduction

Adults living with multiple chronic conditions (MCC) experience a high, overlapping symptom burden (e.g., pain, dyspnea, fatigue, insomnia, anxiety, and depression) that can accelerate functional decline, trigger exacerbations, and drive avoidable emergency and hospital utilization [1, 2, 3]. Yet symptom management is often fragmented across disease‐specific care pathways, creating polypharmacy risk, conflicting recommendations, and care plans that are poorly aligned with what matters most to patients and caregivers [4, 5, 6, 7].

Palliative care offers an evidence‐informed, person‐centered approach to symptom assessment, communication, and goal‐concordant planning, but remains underused or introduced late especially for noncancer MCC trajectories [2, 8, 9]. A scalable solution is needed that fits the realities of primary care, where most MCC management occurs and where workforce limitations make universal specialist palliative care impractical [10, 11, 12, 13].

This paper proposes a practical framework for integrating person‐centered palliative symptom management into routine primary care using standardized symptom screening with the Edmonton Symptom Assessment System (ESAS), structured triage, and stepped escalation to outpatient specialty palliative care when complexity exceeds primary care capacity [2, 7, 8, 12, 14, 15].

### 1.1 Risk Factors for MCC

Persons living with MCC pose a clinical challenge in all areas of healthcare that include acute and chronic care, behavioral health, transitions and coordination of care, self‐management and medication use among others [4]. Disparities associated with MCC are found in racial and ethnic minorities and those who are socioeconomically disadvantaged [4, 7]. These populations experience a higher burden of MCC that occur at an earlier age and with limited access to healthcare placing them at a higher risk for poor quality care [4]. Persons with limited education, low health literacy, cognitive alterations and non‐English speaking are at an even greater risk for a higher burden of care [4, 7].

Persons with MCC experience associated symptoms that if poorly managed lead to disease exacerbation, hospitalization, debility, progressive decline, costly care and poor quality of life [1, 2]. Persons who are at a greater risk for disease exacerbation (e.g., heart failure [HF], chronic obstructive pulmonary disease [COPD]) can benefit from the implementation of concurrent palliative symptom management. Earlier palliative integration can pro‐actively prevent disease exacerbation, maintain physical function, reduce hospitalization and cost of care by promoting patient, family, caregiver wellbeing [1, 2]. Palliative care, with its person‐centered, holistic philosophy, offers a crucial framework for managing symptomatic MCC.

### 1.2 Federal Initiatives in Person‐Centered Care Planning in MCC

AHRQ has been instrumental in maintaining federal momentum to address the complex care needs of the escalating population of persons with MCC. In 2010 HHS released the *Multiple Chronic Conditions: Strategic Framework* with the expectation of implementing strategies that would lend to optimum health and quality of life for persons with MCC [16]. Since the release of the strategic framework AHRQ has developed initiatives, funded research and partnered with other federal agencies to identify gaps in addressing the health status of persons with MCC. AHRQ was one of several federal agencies (e.g., CDC, National Institutes of Health (NIH) etc.) who participated in the HHS Interagency Workgroup on MCC and provided guidance on ensuring a comprehensive effort for achieving the vision of optimal health for this population [16].

AHRQ in 2024–2025 funded and collaborated with academic partners from Oregon Health Science University (OHSU) to initiate person‐centered care planning for persons with or at risk for MCC. This initiative established a learning collaborative of front‐line providers, researchers, organizational leaders, policy makers, patients, families, and caregivers. Following six 3‐h virtual sessions and a cumulative summit in March 2025, content gathered from these meetings was used to develop direction and next steps on best practices to integrate person‐centered care planning for persons with MCC throughout the country [4, 7, 17, 18]. Table 1 identifies components of person‐centered care planning in MCC.

**TABLE 1:** Components of person‐centered care planning in MCC.

Person, family, caregiver, provider	Active collaboration in developing a realistic person‐centered healthcare plan that fits into the person's life. Delineating roles and responsibilities of the team members.
Communication	Shared decision making among all members of the healthcare team with a focus on person preferences. Establish a process for shared communication.
Assessment	Literacy level, physical, behavioral, spiritual and functional status. Identify prioritized problem areas
Determinants of health	Socioeconomic, environmental, occupational, cultural, religious. Access to healthcare and personal safety.
Interventions	Care coordination across the team, choosing interventions that are realistic with consideration of the persons goals and needs. Minimize uncoordinated, unsafe care.
Person‐directed care	Empower the person, family and caregivers in self‐management that follows shared decision making with a focus on readiness to change and ongoing engagement in the care planning.
Monitoring and follow‐up	Evaluation of long‐term engagement in plan of care across all health systems and different providers. Reduction in hospitalization, adverse drug–drug interactions and optimizing physical function.

*Source:* AHRQ [4, 17, 18].

### 1.3 Common Chronic Conditions

In February 2025, President Trump established the *Make America Healthy Again Commission* that includes policy to aggressively combat the escalating rate of mental health disorders, obesity, diabetes, and other chronic conditions [19]. The Administration is focused on chronic disease prevention with many efforts related to children. Although prevention is crucial, effectively managing well‐established MCC remains a public health concern. According to the CDC, over 60% of adults aged 65 years and older are living with cardiovascular disease, diabetes, COPD, chronic kidney disease (CKD), and dementia [20]. Table 2 provides an overview of the ten most common chronic conditions in the U.S. adult population.

**TABLE 2:** Ten most prevalent chronic conditions in the U.S. adult populations.

Rank	Condition	Approximate prevalence	References
1	Obesity	~42%	[21]
2	Hypertension (High blood pressure)	~32%	[21]
3	High total cholesterol (≥ 240 mg/dL)	~11.3%	[22]
4	Hyperlipidemia (elevated LDL/dyslipidemia)	Over 50% with elevated LDL	[23]
5	Diabetes mellitus	~11.6%;	[24]
6	COPD	~6%	[25]
7	Asthma	~8.7%	[26]
8	CKD	~13.9%	[27]
9	Depression (major depressive episodes)	~8.3	[28]
10	Cancer history (any site)	~6.6	[29]

*Source:* Benavidez et al. [20, 30, 31, 32].

Of the most prevalent chronic diseases in the adult population, it is no surprise to find many of these conditions are found in adults living with MCC. The CDC in 2023 released the *Behavioral Risk Factor Surveillance System, 2013–2023* and by tracking disease trends in adults with MCC identified the 10 most common chronic conditions. See Table 3. Select conditions that appear on this list, such as arthritis and hepatitis, are not identified in the 10 most prevalent chronic diseases in adult populations. Arthritis and hepatitis are conditions that are common and interact with the increased disease burden by persons living with MCCs.

**TABLE 3:** Top ten chronic conditions in adults with MCCs.

Rank	Chronic condition
1	Arthritis
2	Asthma
3	Chronic kidney disease
4	Chronic obstructive pulmonary disease (COPD)
5	Cardiovascular disease (heart disease, heart attack, stroke)
6	Cancer (excluding non‐melanoma skin cancer)
7	Depression
8	Diabetes
9	Hypertension
10	Hepatitis

*Source:* CDC [32, 33].

### 1.4 Symptoms and Disease Exacerbations

Persons living with MCC experience significant symptom burden that often limits them from engaging in things that matter most. Individually, most chronic conditions produce similar symptoms such as pain, dyspnea, fatigue, depression, insomnia, and anxiety among others that, if not well managed, can trigger a decline in physical function and increase disease severity [34, 35, 36]. There are, however, individual differences when anticipating symptoms based on disease pathology [37]. See Table 4 for examples of disease‐specific symptoms.

**TABLE 4:** Disease specific symptoms.

Heart failure	Chronic obstructive pulmonary disease	Chronic kidney disease	Alzheimer's dementia
Fatigue	Dyspnea	Pain	Agitation
Edema	Fatigue	Fatigue	Anxiety
Dyspnea	Pain	Pruritus	Pain
Cough	Cough	Numbness	Mood alterations
Numbness	Exercise Intolerance	Insomnia	Insomnia
Insomnia	Insomnia	Depression	Dyspnea
Exercise intolerance			

*Source:* AlHosni et al. [37] and Brooks [38].

According to the *Integrated Model of Multimorbidity and Symptom Science*, the inter‐relatedness between the underlying health condition, symptoms, and disease‐directed therapies all influence patient‐centered outcomes [3]. There is a well‐established link between MCC and an increased risk for acute infections or disease exacerbations due to an altered immune system and reduced physiological reserves [39]. The risk for hospitalization in MCC from an acute exacerbation is increased by 1.35 times for each chronic condition and 1.55 times for each affected body system [40]. The increased risk for hospitalization from HF and COPD from poorly managed symptoms is further amplified by exacerbation [40]. Frequent conditions that contribute to hospitalizations from exacerbation in the U.S. are primarily cardiovascular and pulmonary related. See Table 5.

**TABLE 5:** Conditions responsible for frequent hospitalization.

Rank	Chronic condition	Frequency/readmissions in 2018
1	Septicemia	314 600 readmissions (~8.3% of total)
2	Heart failure	233 100 readmissions (~6.1%)
3	Diabetes mellitus with complication	122 400 readmissions (~3.2%)
4	Chronic obstructive pulmonary disease (COPD) and bronchiectasis	106 300 readmissions (~2.8%)
5	Pneumonia (excluding TB)	97 500 readmissions (~2.6%)
6	Acute and unspecified renal failure	96 900 readmissions (~2.6%)

*Source:* Weiss and Jiang [41].

Frequent hospitalizations for persons with MCC carry far‐reaching physical, emotional, social, and economic consequences. While acute care is sometimes unavoidable, a proactive, coordinated, palliative approach to symptomatic MCC management, transitional care, and patient‐centered care planning is essential in mitigating these effects. Addressing the root causes of rehospitalization and investing in supportive services to maintain independence, improve quality of life, and reduce health system strain and financial burden.

### 1.5 Disease Trajectory

The effective integration of person‐centered palliative symptom management requires an understanding of the disease trajectory and ability to discern the person's own understanding of their underlying condition. This dynamic process requires open communication and interaction between patient, family, caregivers and the interdisciplinary healthcare team to ensure individualized care planning [2]. All too often the clinical attention is focused on the condition with an ominous prognosis or a trajectory with a rapid decline such as cancer and is not all inclusive [2, 42, 43].

**FIGURE 1:**
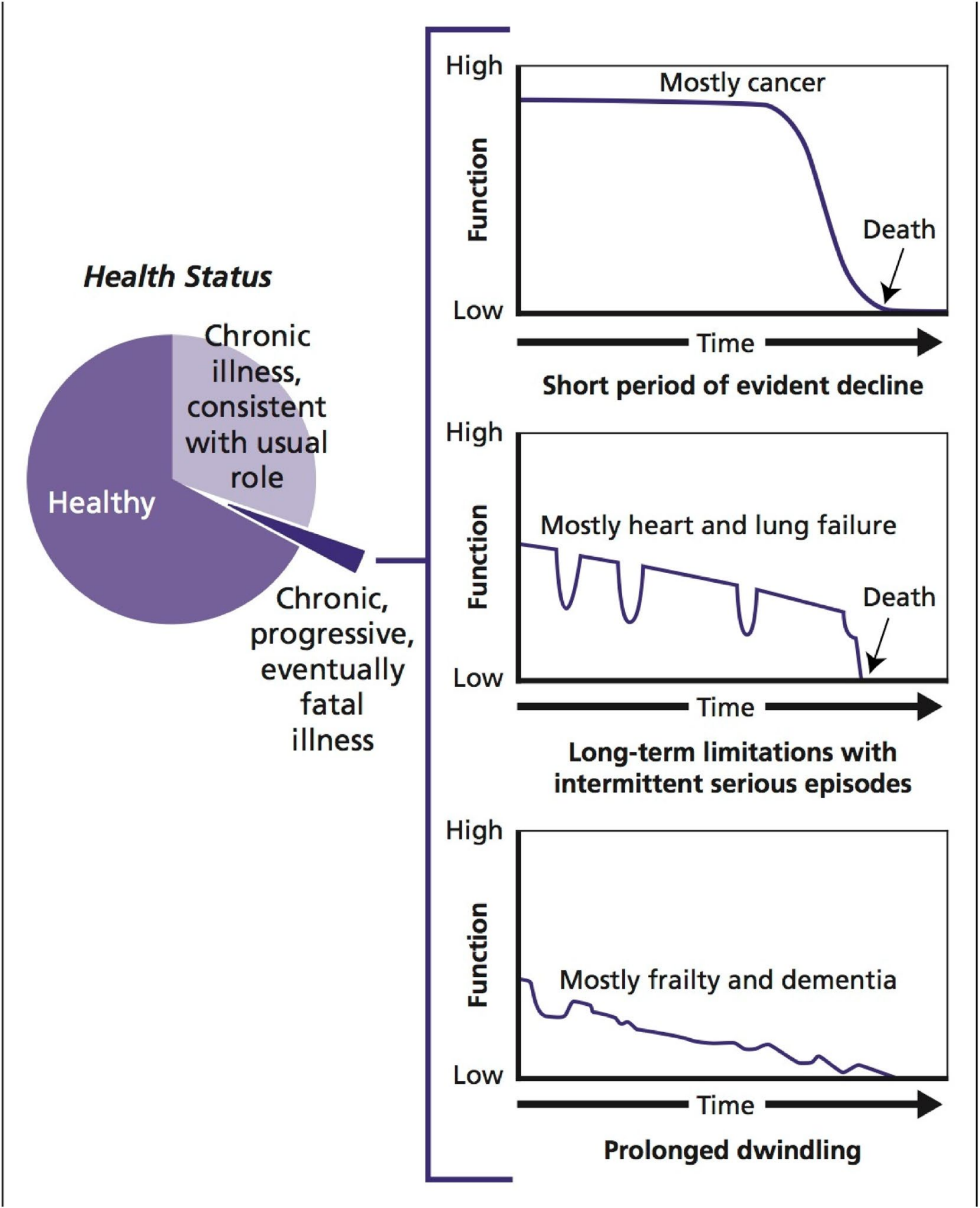
Disease trajectory. Disease Trajectory used with permission from Lynn and Adamson [44] WP‐137 https://www.rand.org/pubs/white_papers/WP137.html.

The Rand Corporation, in 2003, highlighted three unique disease trajectories.

for persons living with chronic conditions (see Figure 1). A short disease trajectory with a sudden progressive decline in physical functioning can be found in cancer. Whereas a longer and slower progressive disease trajectory occurs in HF and COPD, these conditions are mixed with intermittent bouts of disease exacerbation, hospitalization and recovery [35, 44, 45]. A prolonged dwindling, insidious trajectory with progressive fragility occurs in persons diagnosed with Alzheimer's or dementia [44, 45].

The onset of a disease trajectory occurs once a person is diagnosed with a non‐reversible condition such as HF or COPD and is aligned with a prognosis [46]. Determining a prognosis helps to better understand the likely course of disease, while the trajectory of disease is focused on changes in physical functioning, overall health status, and person‐centered quality of life can be evaluated over a timeframe [45, 47]. According to the [48] *Guiding Principles on the Care of Older Adults with MCC*, gaining an understanding of the disease trajectory and prognosis allows for shared decision making between the person, family, caregiver, and provider. Developing a person‐centered care plan should include four key components that align with the AHRQ [4] and Institute for Healthcare Improvement's age‐friendly care [49] recommendations:

- Allow for open communication between the person and all members of the healthcare team to develop and align a care plan that is focused on the person's health priorities.
- Determine the intensity of interventions based upon the person's decision to initiate or discontinue aggressive or non‐aggressive therapies.
- Assess and evaluate the ongoing benefit versus harm or any associated burden of care that comes from disease management.
- Implementing the 4Ms, what matters most to the person, use of appropriate medications, maintaining mentation and mobility [49].

It is important to monitor, assess, and evaluate changes in prognosis through appropriate diagnostics (e.g., ejection fraction, pulmonary function, radiographic findings etc.), appropriate medication management and the degree of physical functioning [1, 4]. These are individualized considerations when developing a person‐centered care plan that optimizes health outcomes [4]. Person‐centered care planning requires a thorough, multidimensional assessment of the person and family. Care planning requires personalization and should consider sources of distress and the influence of the personal, family, cultural and spiritual context for that person [42]. Ongoing updates to the care‐planning should correlate with evolving and dynamic therapeutic options [2].

### 1.6 Edmonton Symptom Assessment System

The ESAS was developed with a specific purpose of rapidly screening several symptoms at once in daily clinical practice [14]. After its initial application it became clear that the clinical information gained from ESAS was useful for providers and was not a burden for persons to complete at each encounter [14]. ESAS consists of 10 symptoms (pain, fatigue, appetite, dyspnea, nausea, depression, anxiety, wellbeing, drowsiness, night sleep) and is measured from a numerical scale of 0 (no symptom) to 10 (worst possible symptom) over the previous 24 h. This tool can be used by paper, electronic, or by telephone.

The ESAS was developed completely free from copyright so that users anywhere in the world could assess and evaluate persons with symptomatic conditions without barriers. Over time it was translated to more than sixty different languages [15] and has been adopted as a standard clinical assessment tool. ESAS was independently validated by many research teams and published in multiple peer‐reviewed journals. One of its main advantages is that it allows multidimensional assessment in one minute or less and most of the time it can be completed by a person alone or with a family member [50]. The simplicity of ESAS has made it easy for adoption in a busy clinical setting and especially in low‐ and middle‐income countries where resources are limited [15].

In recent years two additional dimensions have been added to the original ten symptoms and include financial and spiritual distress. These newer dimensions are not part of the validated total symptom distress score and the physical and psychosocial sub scores of the test, but they provide valuable and actionable information that can be used for supporting the person who may be experiencing stress and worry. The opportunity to repeatedly screen for multiple symptoms has not only allowed the deployment of clinical teams to address the person's specific needs but has allowed the voice of the person to be heard [14] (Table 6).

**TABLE 6:** Edmonton symptom assessment system.

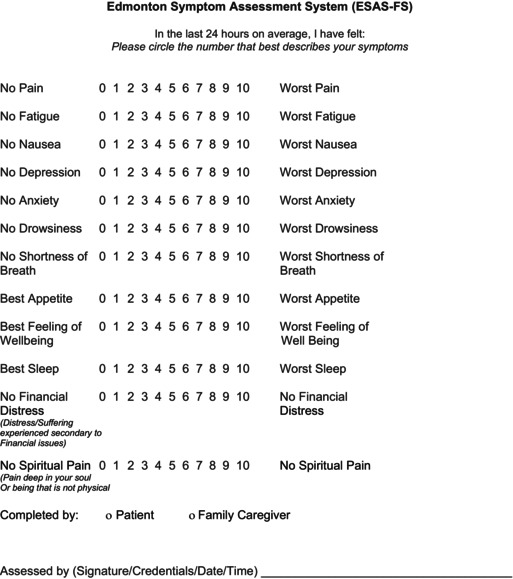

### 1.7 Paradigm Shift in Palliative Care

The WHO [51], the NASEM [10], and HHS [11] have recommended that palliative care be integrated upstream into primary care. These entities released reports and publications that suggest a need to focus on the broader determinants of health by addressing the comprehensive and interrelatedness of a person's physical, mental, spiritual, and social dimensions through palliative person‐centered care [10, 11, 42, 51].

The WHO suggests that primary care offers whole‐person care throughout the person's lifespan to include quality palliative care which can be integrated into person‐centered care planning for those living with MCC [51]. The NASEM reports there are shared principles between primary and palliative care that are person and family centered. Health care that is continuous, comprehensive, and equitable—team‐based care that is collaborative, coordinated, integrated, accessible, and of high quality [10].

A recent consensus publication by members of the American Academy of Nursing (AAN) expert panels on palliative and end of life care, primary care, aging, acute and critical care, and two expert physicians embrace these recommendations. The authors highlight how the integration of palliative symptom management can improve person‐centered care, be cost effective, and support the need for coordinated, complex, comprehensive care [2]. Utilizing person‐centered care planning allows the focus of care on the person's values and preferences while including the family and caregivers [4, 12].

Primary and palliative person‐centered care share the same principles. These principles include health promotion, disease and symptom prevention, curative care, rehabilitation, and continuity of care [2, 9, 10, 13]. The WHO agrees that specialists who provide palliative care are not always necessary when meeting the palliative care needs of most persons and their families in the primary care setting. The WHO further suggests palliative care be provided by general practitioners, family physicians, and advanced practice providers within the community [13].

The NASEM [10] report states there are not enough palliative specialists to meet the advanced care needs of the nation's escalating older population living with MCCs. Persons at risk or living with MCC often require aggressive symptom management and moving palliative care upstream into the primary care setting versus reserving it for the end‐of‐life will promote comprehensive, coordinated, and preventative person‐centered care. Persons living with MCC are not terminally ill and often live for several years following a diagnosis of a non‐reversible condition.

### 1.8 Stepped Integration of Primary Versus Specialty Outpatient Palliative Care in MCC: Who, When, Where, How

The escalating prevalence of symptomatic MCC requires a practical outpatient model that can expand access to palliative symptom management without overwhelming a limited specialty workforce. In primary care, most adults with MCC already receive longitudinal disease management and care coordination; however, symptom burden, polypharmacy, and competing guideline recommendations frequently exceed the capacity of traditional disease‐focused visits. A stepped‐care approach provides a pragmatic bridge: primary care teams already deliver palliative care (standardized symptom screening, initial symptom protocols, and person‐centered care planning), while outpatient specialty palliative care is reserved for persons with persistent high symptom distress, rapid functional decline, complex decision‐making, or caregiver strain. Stepped palliative care has been tested as a strategy to preserve patient‐centered outcomes while using fewer specialty resources, supporting its relevance as a model to translate into MCC trajectories where symptom burden is common and episodic exacerbations drive utilization [52].

#### 1.8.1 Step 1: Universal Palliative Care in Primary Care (Default for MCC)

Primary care teams can normalize symptom assessment as a routine vital sign by deploying brief, standardized tools such as the ESAS that capture multiple symptom domains quickly and can be administered on paper, electronically, or by telephone [14, 15]. ESAS‐supported review creates a shared language between the person, caregiver, and clinical team and supports consistent follow‐up over time, including rapid multidimensional reassessment [15, 50]. For most persons with MCC, Step 1 includes: (1) symptom identification and prioritization (e.g., pain, dyspnea, fatigue, insomnia, depression and anxiety), (2) a short protocolized first‐line management plan aligned with disease status and patient goals, (3) medication reconciliation focused on symptom‐relieving and harm‐reducing regimens, and (4) person‐centered care planning that integrates values, tradeoffs, function, and caregiver context [2, 42]. This step operationalizes upstream palliative care in the same setting where MCC care is already delivered [2, 51].

#### 1.8.2 Step 2: Targeted Consultative Palliative Support (Intermediate Intensity)

A subset of persons with MCC will require additional support beyond Step 1, but not necessarily longitudinal specialty palliative care. Step 2 emphasizes right‐sized assistance, including e‐consultation, tele‐palliative co‐management visits, brief interdisciplinary case review (e.g., social work, chaplaincy), or time‐limited specialty input focused on a specific symptom cluster (e.g., refractory dyspnea with anxiety; complex pain with renal impairment; delirium risk and caregiver burnout) [2, 8]. Evidence from ambulatory models and primary‐care–relevant identification approaches indicates that outpatient integration strategies can strengthen supportive care processes including proactive needs identification, structured follow‐up, and communication when implemented with clear criteria and escalation pathways [8, 35, 36]. Step 2 is particularly useful for primary care practices serving high‐need populations, where limited visit time and fragmented specialty access can delay symptom stabilization [2].

#### 1.8.3 Step 3: Specialty Outpatient Palliative Care (Highest Intensity; Ongoing Co‐Management When Needed)

Specialty outpatient palliative care is indicated when symptom complexity, psychosocial‐spiritual distress, decision conflict, or caregiver strain exceeds what primary care can safely provide. Timely palliative referral frameworks emphasize standardized referral criteria to reduce variability and late referrals; this principle translates well to MCC, where a prognosis‐based referral is often less useful than a needs‐based referral. Specialty outpatient palliative care contributes unique value through advanced symptom pharmacology (including opioid stewardship and adjuvant titration), deprescribing and interaction management across multiple conditions, structured goals‐of‐care communication during transitions, and interdisciplinary supports that address caregiver distress and social determinants of health [42].

#### 1.8.4 Who Should Be Considered (Needs‐Based Triggers)

To make the transition from Step 1, 2 to Step 3 actionable, practices should adopt simple triggers tied to symptom burden, utilization, and complexity. Examples include: persistent moderate‐to‐severe symptom scores across ≥ 2 encounters despite protocolized management (e.g., repeated high ESAS dyspnea, pain, fatigue, depression and anxiety); recurrent exacerbations, frequent emergency visits, or hospitalizations for cardiopulmonary or infection‐related events; rapid functional decline, frailty transitions, or increased physical dependence with safety concerns; complex polypharmacy with conflicting guideline recommendations (e.g., heart failure, CKD, COPD, and depression) where harm minimization becomes a dominant goal; caregiver strain that is impairing home management; and high‐stakes decisions (e.g., device therapy in advanced heart failure, feeding decisions in dementia, or repeated hospital admissions with diminishing recovery). In MCC, these triggers often cluster, and the purpose of stepped care is to escalate early enough to prevent destabilizing cycles of exacerbation and hospitalization [2, 42].

#### 1.8.5 When and How the Transition Occurs (Workflow)

The transition should be framed as a *shared‐care intensification* rather than a handoff away from primary care. A practical workflow is: (1) screen symptoms implementing the ESAS at defined intervals and during exacerbations; (2) initiate Step 1 protocols and document a person‐centered care plan; (3) reassess within a defined timeframe (e.g., 2–6 weeks depending on severity) and, if triggers persist, activate Step 2 supports; (4) if triggers continue or safety and complexity increases, place an outpatient palliative care referral with a warm handoff that includes the current care plan, symptom trajectory, medication list with prior trials, and stated person goals and values. Follow‐up intensity can then be dosed according to need and organizing principle consistent with stepped palliative care models so that those with lower needs receive lighter‐touch follow‐up while those with higher needs receive more frequent specialty contact [42, 52].

#### 1.8.6 Where This Occurs (Outpatient Delivery Options)

Outpatient palliative integration can be delivered through clinic‐based models (primary care, federal qualified health clinics, geriatric chronic disease clinics or long‐term care) embedded interdisciplinary support, and telehealth‐enabled palliative visits. Comparative effectiveness evidence supports that early palliative care delivered via secure video can achieve outcomes equivalent to in‐person delivery in serious illness contexts, strengthening feasibility for rural or access‐limited settings [42, 53, 54, 55]. For MCC, telehealth and telephone follow‐up can be particularly useful for symptom monitoring, caregiver coaching, and medication titration between visits, while in‐person encounters can be reserved for complex physical assessments and major care planning conversations.

In summary, stepped outpatient palliative integration aligns palliative symptom management with the realities of MCC: it is needs‐based not prognosis‐dependent, primary‐care‐anchored, protocolized, and scalable. It also creates clear who, when, how criteria for transitioning from primary palliative care to specialty outpatient palliative care while maintaining continuity through shared person‐centered care planning and iterative reassessment.

### 1.9 Guideline‐Informed Symptom Management in MCC: How to Adapt Disease‐Specific Recommendations Using a Stepped Outpatient Approach

A guideline‐informed crosswalk of common symptom management and strategies that frequently arise in MCC are highlighted in Table 7. This table critically highlights MCC‐specific adaptation considerations (e.g., renal and hepatic dosing constraints, hypotension and fall risk, cognitive vulnerability, and drug–drug interactions) to support safe application in primary care and to inform stepped escalation to outpatient specialty palliative care when complexity exceeds primary care capacity.

**TABLE 7:** Guideline‐informed symptom management in MCC: How to adapt disease‐specific recommendations using a stepped outpatient approach.

Symptom/need (ESAS domain)	Disease‐specific guideline anchor (examples)	MCC adaptation considerations (what changes in multimorbidity)	Primary care palliative actions (Step 1–2)	Escalation to specialty outpatient palliative care (Step 3)
Dyspnea	HF guideline‐directed therapy; COPD inhaled bronchodilation + pulmonary rehab	HF/COPD overlap; anxiety‐dyspnea cycle; renal dosing limits; avoid oversedation in COPD/OSA; inhaler technique failures common	Optimize volume status + inhalers; fan/positioning; pulmonary rehab referral; treat anxiety; consider low‐dose opioid for refractory dyspnea when appropriate	Persistent severe dyspnea despite optimization; repeated exacerbations/ED use; need for complex opioid titration; dyspnea with significant existential/spiritual distress
Pain (nociceptive/neuropathic)	CKD neuropathy guidance; dementia pain recognition; osteoarthritis strategies	Avoid/limit NSAIDs in CKD/HF; opioid sensitivity in older adults; drug–drug interactions (CNS depressants); fall risk	Acetaminophen/topicals; nonpharmacy (PT, heat/ice); neuropathic agents with cautious dosing; structured opioid risk/benefit discussion when needed	Pain ≥ moderate–severe despite ≥ 2 trials; complex opioid rotation; co‐occurring depression/substance risk; significant functional decline tied to pain
Fatigue/exercise intolerance	HF/cardiac rehab; COPD pulmonary rehab	Often multifactorial (sleep, anemia, depression, meds, deconditioning); competing activity limits across conditions	Medication review (sedatives, beta‐blocker burden, anticholinergics); sleep optimization; pacing/energy conservation; rehab referral; address depression	Rapid functional decline; severe fatigue limiting ADLs despite optimization; unclear drivers requiring interdisciplinary assessment
Anxiety/depression	COPD/HF symptom burden literature; dementia caregiver stress	Avoid polypharmacy; watch QT risk, hyponatremia; cognitive impairment may mask mood symptoms; caregiver distress often co‐present	Brief counseling + behavioral activation; SSRI/SNRI when appropriate; social work referral; caregiver screening/support; address financial/spiritual distress	Suicidality; severe anxiety driving dyspnea/panic; complicated grief/moral distress; caregiver burnout threatening home safety
Insomnia/sleep disturbance	Dementia behavioral approaches: chronic illness sleep hygiene	Avoid benzodiazepines/Z‐drugs in falls/COPD/OSA; anticholinergic burden worsens cognition	Sleep hygiene + CBT‐I resources; melatonin when appropriate; deprescribe stimulants/sedatives; treat nocturia/pain	Refractory insomnia with delirium risk; need for complex deprescribing plan; nighttime agitation with safety concerns
Appetite/weight loss/nausea	HF cachexia considerations; COPD nutrition; CKD dietary limits	Conflicting dietary prescriptions (HF sodium, CKD potassium/phosphate, diabetes carbs); med adverse effects; constipation‐driven nausea common	Identify reversible causes; bowel regimen; antiemetic trial; nutrition consults to reconcile competing diets around patient goals	Persistent nausea/weight loss with dehydration; feeding/aspiration decisions; high decision conflict about tradeoffs
Pruritus (common in CKD)	CKD‐associated pruritus guidance	Renal dosing for gabapentinoids; xerosis; drug triggers; sleep disruption feeds fatigue/mood symptoms	Emollients/skin care; review triggers; cautious gabapentin/pregabalin; sleep support	Severe pruritus impairing function/sleep despite trials; complex medication intolerance requiring specialist titration
Cognitive impairment/delirium risk/agitation	Dementia evaluation/behavioral management guidance	High anticholinergic burden; infection/med changes trigger delirium; caregiver capacity determines safety	Screen for delirium triggers; simplify regimen; nonpharmacy routines; caregiver coaching; safety planning	Severe agitation/safety risk; recurrent delirium; major goals‐of‐care decisions (driving, placement, feeding, hospitalization tradeoffs)
Caregiver strain + goals‐of‐care alignment	Serious illness communication frameworks	MCC trajectories fluctuate; decisions recur after each exacerbation; discordant specialist advice is common	Document values/goals; clarify tradeoffs; advance care planning; coordinate across specialties; identify surrogate decision maker	Persistent decision conflict; repeated hospitalizations with diminishing recovery; complex tradeoffs (devices, dialysis escalation, repeated ICU admissions)

While many symptom recommendations originate from single‐condition guidelines, MCC care requires clinicians to reconcile competing priorities, balance harms and benefits across conditions, and align interventions with what matters most to the person and caregiver. In MCC, symptoms often share overlapping mechanisms and are shaped by therapies for co‐occurring diseases; therefore, symptom management should begin with ESAS identifying explicit symptom prioritization and intentional review of medication burden and interaction risk. Table 7 is designed to be used as a practical translation aid rather than an exhaustive guideline summary assisting primary care teams in identifying a reasonable first‐line approach, anticipate MCC caveats that commonly derail symptom control (e.g., renal function, sedation risk, delirium risk), and document a person‐centered plan that can be revisited iteratively over time. Importantly, Table 7 intentionally pairs disease‐based anchors with MCC adaptation prompts because the evidence base remains limited for how best to manage symptoms when conditions and treatments interact synergistically or antagonistically. The interrelatedness between underlying conditions, symptoms, and disease‐directed therapies influences patient‐centered outcomes and can accelerate exacerbations when symptoms are not managed proactively.

Accordingly, the MCC adaptation column emphasizes harm‐minimization (e.g., avoiding medication classes likely to worsen co‐morbid disease states), deprescribing where appropriate, and early identification of complexity thresholds that should trigger Step 2 consultative support or Step 3 outpatient specialty palliative care (e.g., persistent moderate–severe symptom distress across encounters despite protocolized management, recurrent utilization, or high decision conflict). This approach clarifies how to apply disease‐specific recommendations within MCC realities while also highlighting where future research is needed to strengthen evidence for multimorbidity‐tailored symptom management.

## 2 Conclusion

Person's living with or at risk for MCC represent a vulnerable population who experience significant physical, emotional, social, and spiritual symptoms. The traditional disease‐focused model is inadequate to meet their complex care needs, and reserving palliative interventions for the end‐of‐life prevents optimizing quality symptom management throughout the disease trajectory. An approach rooted in the principles of palliative care with a person‐centered focus is essential. By systematically integrating palliative symptom management earlier in the course of the disease trajectory, the healthcare system can move beyond simply managing chronic conditions to truly promoting person‐centered care planning by ensuring comprehensive primary care that prevents disease exacerbation, improves quality of life, preserves dignity, and allows the person to focus on what matters most.

Future directions should include implementation, research, training models for primary care teams, demonstrate current use of reimbursement mechanisms, and develop quality outcome measures.

## Conflicts of Interest

The authors declare no conflicts of interest.

## Data Availability

The data that support the findings of this study are available from the corresponding author upon reasonable request.

## References

[1] K. Kuebler , “Palliative Care for Symptomatic Heart Failure: A New Paradigm,” Journal for Nurse Practitioners 19, no. 2 (2023): 104472, 10.1016/j.nurpra.2022.10.001.

[2] K. Kuebler , T. Monroe , R. Ricciardi , et al., “Integration of Palliative Care in the Management of Multiple Chronic Conditions: An Expert Consensus Paper With Policy Implications,” Nursing Outlook 72, no. 6 (2024): 102273, 10.1016/j.outlook.2024.102273. 39388799 PMC11611613

[3] T. Tripp‐Reimer , J. K. Williams , S. E. Gardner , et al., “An Integrated Model of Multimorbidity and Symptom Science,” Nursing Outlook 68, no. 4 (2020): 430–439. 32482344 10.1016/j.outlook.2020.03.003 PMC7483649

[4] Agency for Healthcare Research and Quality , “Advancing Patient‐Centered Care for People With Multiple Chronic Conditions,” (2024) accessed July 15, 2026, https://www.ahrq.gov/patient‐safety/settings/long‐term‐care/resource/multichronic/mcc.html.

[5] A. Bierman , “AHRQ Unveils Research Agenda to Transform Care for People With Multiple Chronic Conditions,” (2021) accessed July 15, 2026, https://www.ahrq.gov/news/blog/ahrqviews/transform‐care‐mcc.html.

[6] A. Bierman , “Caring for People With Multiple Chronic Conditions: Health Systems Transformation Improving Care for Persons at Risk or Living With Multiple Chronic Conditions,” (2025) Unpublished slide presentation summary of learning collaborative.

[7] B. Watson , L. Estenson , A. Eden , et al., “Person‐Centered Care Planning for People Living With or at Risk for Multiple Chronic Conditions,” Journal American Medical Association Network Open 7, no. 10 (2024): e2439851, 10.1001/jamanetworkopen.2024.39851. PMC11581598 39418021

[8] D. Hui , Y. Heung , and E. Bruera , “Timely Palliative Care: Personalizing the Process of Referral,” Cancers 14, no. 4 (2022): 1047, 10.3390/cancers14041047. 35205793 PMC8870673

[9] S. Wy , J. Waldfogel , D. Sloan , et al., Integrating Palliative Care in Ambulatory Care of Noncancer Serious Chronic Illness: A Mixed‐Methods Review. Comparative Effectiveness Review No. 237. AHRQ Publication No. 21‐EHC002 (Agency for Healthcare Research and Quality, 2021), 10.23970/AHRQEPCCER237.

[10] National Academies of Sciences, Engineering, and Medicine , Integrating Serious Illness Care Into Primary Care Delivery: Proceedings of a Workshop (National Academies Press, 2022), 10.17226/26411. 35471810

[11] U.S. Department of Health and Human Services , “HHS is Taking Action to Strengthen Primary Care,” (2023) accessed July 15, 2026, https://www.hhs.gov/sites/default/files/primary‐care‐issue‐brief.pdf.

[12] World Health Organization , “Integrating Palliative Care and Symptom Relief Into Primary Health Care: A WHO Guide for Planners, Implementers and Managers,” (2018) accessed July 15, 2026, https://www.who.int/publications/i/item/integrating‐palliative‐care‐and‐symptom‐relief‐into‐primary‐health‐care.

[13] World Health Organization , “Why Palliative Care is an Essential Function of Primary Care,” (2018) accessed July 15, 2026, https://www.who.int/docs/default‐source/primary‐health‐care‐conference/palliative.pdf.

[14] E. Bruera , N. Kuehn , M. J. Miller , P. Selmser , and K. Macmillan , “The Edmonton Symptom Assessment System (ESAS): A Simple Method for the Assessment of Palliative Care Patients,” Journal of Palliative Care 7, no. 2 (1991): 6–9. 1714502

[15] D. Hui and E. Bruera , “The Edmonton Symptom Assessment System 25 Years Later: Past, Present, and Future Developments,” Journal of Pain and Symptom Management 53, no. 3 (2016): 630–643, 10.1016/j.jpainsymman.2016.10.370. 28042071 PMC5337174

[16] U.S. Department of Health and Human Services , “Multiple Chronic Conditions a Strategic Framework,” (2010) accessed July 15, 2026, https://www.hhs.gov/sites/default/files/ash/initiatives/mcc/mcc_framework.pdf.

[17] Agency for Healthcare Research and Quality , “Summit on Transforming Care for People Living With Multiple Chronic Conditions,” (2020), accessed July 15, 2026, https://www.ahrq.gov/sites/default/files/wysiwyg/patient‐safety/settings/mcc‐summit/mcc‐summit‐proceedings.pdf.

[18] Agency for Healthcare Research and Quality , “Multiple Chronic Conditions,” (2023), accessed July 15, 2026, https://www.ahrq.gov/patient‐safety/settings/long‐term‐care/resource/multichronic/mcc.html.

[19] The White House , “Establishing the Presidents Make America Healthy Again Commission,” (2025) accessed July 15, 2026, https://www.whitehouse.gov/presidential‐actions/2025/02/establishing‐the‐presidents‐make‐america‐healthy‐again‐commission/.

[20] G. Benavidez , W. Zahnd , P. Hung , and J. Eberth , “Chronic Disease Prevalence in the US: Sociodemographic and Geographic Variations by Zip Code Tabulation Area,” Preventing Chronic Disease 21 (2024): 230267. 10.5888/pcd21.230267 PMC10944638 38426538

[21] I. Telefords , M. McCGough , D. Tevis , and L. Cotter , “How has the Burden of Chronic Diseases in the U.S. and Peer Nations Changed Over Time?—Peterson‐KFF Health System Tracker Peterson‐KFF Health System Tracker,” (2025).

[22] M. Carroll , C. Fryar , J. Gwira , and M. Iniguez , Total and High‐Density Lipoprotein Cholesterol in Adults: United States, August 2021–August 2023 (NCHS Data Brief No. 515), ed. National Center for Health Statistics (Centers for Disease Control and Prevention (CDC) Stacks, 2024), https://stacks.cdc.gov/view/cdc/165796. 10.15620/cdc/165796 PMC11726265 39750952

[23] Y. Gao , L. Shah , J. Ding , and S. Martin , “US Trends in Cholesterol Screening, Lipid Levels, and Lipid‐Lowering Medication Use in US Adults, 1999 to 2018,” Journal of the American Heart Association 12, no. 3 (2023): e028205, 10.1161/JAHA.122.028205. 36625302 PMC9973640

[24] Centers for Disease Control and Prevention , National Diabetes Statistics Report (U.S. Department of Health and Human Servicess, 2024), https://www.cdc.gov/diabetes/php/data‐research/index.html.

[25] Y. Liu , S. Carlson , K. Watson , F. Xu , and K. J. Greenlund , “Trends in the Prevalence of Chronic Obstructive Pulmonary Disease Among Adults Aged ≥ 18 Years United States, 2011–2021,” Morbidity and Mortality Weekly Report 72, no. 46 (2023): 1250–1256. 37971940 10.15585/mmwr.mm7246a1 PMC10684355

[26] American Lung Association , “Current Asthma Demographics,” (2025) accessed 15 July, 2026, https://www.lung.org/research/trends‐in‐lung‐disease/asthma‐trends‐brief/current‐demographics.

[27] Centers for Disease Control and Prevention , Fast Facts: Health and Economic Costs of Chronic Conditions (U.S. Department of Health and Human Services, 2024), https://www.cdc.gov/chronic‐disease/data‐research/facts‐stats/index.html.

[28] National Institute of Mental Health , Major Depression (U.S. Department of Health and Human Services, National Institutes of Health, 2023), https://www.nimh.nih.gov/health/statistics/major‐depression.

[29] Centers for Disease Control and Prevention , History of Cancer (National Center for Health Statistics, 2023), https://www.cdc.gov/nchs/hus/topics/history‐of‐cancer.htm.

[30] Centers for Disease Control and Prevention , “Prevalence of Multiple Chronic Conditions Among US Adults, 2018,” (2020), accessed July 15, 2026, https://www.cdc.gov/pcd/issues/2020/20_0130.htm.

[31] Centers for Disease Control and Prevention , “About Chronic Diseases,” (2022), accessed July 15, 2026, https://www.cdc.gov/chronicdisease/about/index.htm.

[32] Centers for Medicare and Medicaid , “Multiple Chronic Conditions,” (2021), accessed July 15, 2026, https://www.cms.gov/Research‐Statistics‐Data‐and‐Systems/Statistics‐Trends‐and‐Reports/Chronic‐Conditions/MCC_Main.

[33] Centers for Disease Control and Prevention , “Trends in Multiple Chronic Conditions Among US Adults, by Life Stage, Behavioral Risk Factor Surveillance System, 2013–2023,” (2023) accessed July 15, 2026, https://www.cdc.gov/pcd/issues/2025/24_0539.htm. 10.5888/pcd22.240539 PMC12007472 40245168

[34] N. Dudley , S. Ritchie , M. Wallhagen , et al., “Characteristics of Older Adults in Primary Care Who May Benefit From Primary Palliative Care in the U.S.,” Journal of Pain and Symptom Management 55, no. 2 (2017): 217–225, 10.1016/j.jpainsymman.2017.09.002. 28916294

[35] Y. ElMokhallalati , S. Bradley , E. Chapman , et al., “Identification of Patients With Potential Palliative Care Needs: A Systematic Review of Screening Tools in Primary Care,” Palliative Medicine 34, no. 8 (2020): 989–1005, 10.1177/0269216320929552. 32507025 PMC7388141

[36] B. A. Graney , D. H. Au , A. E. Barón , et al., “Advancing Symptom Alleviation With Palliative Treatment (ADAPT) Trial to Improve Quality of Life: A Study Protocol for a Randomized Clinical Trial,” Trials 20, no. 1 (2019): 355, 10.1186/s13063-019-3417-1. 31196156 PMC6567600

[37] F. AlHosni , M. Al Qadire , O. A. Omari , H. Al Raqaishi , and A. Khalaf , “Symptom Prevalence, Severity, Distress, and Management Among Patients With Chronic Diseases,” BMC Nursing 22, no. 1 (2023): 155, 10.1186/s12912-023-01296-8. 37149599 PMC10162903

[38] M. Brooks , First Alzheimer's Guideline for Clinical Practice Released (Medscape, 2018), https://www.medscape.com/viewarticle/899674.

[39] Y. Luo , Y. Chen , K. Wang , et al., “Associations Between Multimorbidity and Frailty Transitions Among Older Americans,” Journal of Cachexia, Sarcopenia and Muscle 14, no. 2 (2023): 1075–1082, 10.1002/jcsm.13197. 36852679 PMC10067509

[40] I. Dantas , R. Santana , J. Sarmento , and P. Aguiar , “The Impact of Multiple Chronic Diseases on Hospitalizations for Ambulatory Care Sensitive Conditions,” BMC Health Services Research 16 (2016): 348, 10.1186/s12913-016-1584-2. 27488262 PMC4973077

[41] A. Weiss and H. Jiang , “Overview of Clinical Conditions With Frequent and Costly Hospital Readmissions by Payer, 2018 (Statistical Brief #278). Agency for Healthcare Research and Quality,” (2021) accessed July 15, 2026, https://hcup‐us.ahrq.gov/reports/statbriefs/sb278‐Conditions‐Frequent‐Readmissions‐By‐Payer‐2018.jsp. 34460186

[42] E. Bruera , Some Notes for Physicians Contemplating Career in Palliative Person‐Centered Care, 2nd ed. (University of Texas: MD Anderson Cancer Center, 2024).

[43] A. Bierman and R. Werner , “Special Issue: The Science of Care for People With Multiple Chronic Conditions,” Health Services Research 56, no. S1 (2021): 965–1079.

[44] J. Lynn and D. Adamson , Living Well at the End of Life: Adapting Health Care to Serious Chronic Illness in Old Age (Rand Corporation, 2003), 10.7249/wp137.

[45] S. Murray , K. Boyd , S. Moine , et al., “Using Illness Trajectories to Inform Person‐Centered, Advance Care Planning,” British Medical Journal 384 (2024): e067896, 10.1136/bmj-2021-067896. 38428953

[46] Global Initiative for Chronic Obstructive Pulmonary Disease , “Global Strategy for Prevention, Diagnosis and Management of COPD,” (2025) accessed July 15, 2026, https://goldcopd.org/2025‐gold‐report/.

[47] C. Boyd , C. D. Smith , F. A. Masoudi , et al., “Decision Making for Older Adults With Multiple Chronic Conditions: Executive Summary for the American Geriatric's Society Guiding Principles on the Care of Older Adults With Multimorbidity,” Journal of the American Geriatrics Society 67, no. 4 (2019): 665–673, 10.1111/jgs.15809. 30663782

[48] American Geriatric Society , “Guiding Principles for the Care of Older Adults With Multimorbidity: A Stepwise Approach for Clinicians,” (2012) accessed July 15, 2026, https://geriatricscareonline. org/ProductAbstract/guiding‐principles‐for‐the‐care‐of‐older‐adults‐with‐ multimorbidity/CL012.

[49] Institute for Healthcare Improvement , “Age Friendly Health Systems,” (2025) accessed July 15, 2026, https://www.ihi.org/partner/initiatives/age‐friendly‐health‐systems.

[50] A. Wong , S. Tayjasanant , A. Rodriguez‐Nunez , et al., “Edmonton Symptom Assessment Scale Time Duration of Self‐Completion Versus Assisted Completion in Patients With Advanced Cancer: A Randomized Comparison,” Oncologist 26, no. 2 (2021): 165–171, 10.1002/onco.13619. 33252169 PMC7873322

[51] World Health Organization , “Quality Health Services and Palliative Care: Practical Approaches and Resources to Support Policy, Strategy & Practice,” (2021) accessed July 15, 2026, https://www.who. int/publications/i/item/9789240035164.

[52] J. Temel , V. Jackson , A. El‐Jawahri , et al., “Stepped Palliative Care for Patients Wth Advanced Lung Cancer: A Randomized Clinical Trial,” JAMA 332, no. 6 (2024): 471–481, 10.1001/jama.2024.10398. 38824442 PMC11145511

[53] L. Wang , Delivering Palliative Care by Telehealth Meets the Needs of People With Cancer (National Cancer Institute, 2024).

[54] Administration for Community Living , “ACL Releases New Briefing on Educating Adults About Chronic Disease Self‐Management,” (2017), accessed July 15, 2026, https://acl.gov/news‐and‐events/announcements/acl‐releases‐new‐briefing‐educating‐adults‐about‐chronic‐disease‐self.

[55] Administration for Community Living , “2020 Profile Of Older Americans,” (2021), accessed July 15, 2026, https://acl.gov/sites/default/files/Profile%20of%20OA/2021%20Profile%20of%20OA/2021ProfileOlderAmericans_508.pdf.

